# miR-181a is a negative regulator of GRIA2 in methamphetamine-use disorder

**DOI:** 10.1038/srep35691

**Published:** 2016-10-21

**Authors:** Kai Zhang, Qingzhong Wang, Xuxiu Jing, Yan Zhao, Haifeng Jiang, Jiang Du, Shunying Yu, Min Zhao

**Affiliations:** 1 Collaborative Innovation Center for Brain Science, Shanghai Mental Health Center, Shanghai Jiao Tong University School of Medicine, 600 Wan Ping Nan Road, Shanghai 200030, China; 2 Wuxi Mental Health Center, Nanjing Medical University, 156 Qian Rong Road, Wuxi 214151, China; 3 Shanghai Key Laboratory of Psychotic Disorders, Shanghai, PR China; 4 Brain Science and Technology Research Center, Shanghai Jiao Tong University Shanghai, PR China

## Abstract

A previous study reported that the miR-181a level in serum was significantly different between patients with methamphetamine-use disorder and healthy controls and that chronic methamphetamine use down-regulates the expression of miR-181a. Bioinformatic analysis predicted that miR-181a might bind the 3′-UTRs of the mRNA transcripts of the human glutamate receptor genes GRIA2 and GABRA1. In this study, we measured the expression of GRIA2 and GABRA1 in patients with methamphetamine-use disorder. In addition, we examined whether miR-181a down-regulates GRIA2 and GABRA1 in a cell-based assay. We further examined the effects of chronic methamphetamine exposure on the expression of miR-181a, GRIA2 and GABRA1. The results demonstrated that serum GRIA2 is higher in patients with methamphetamine-use disorder than in healthy controls. Dual luciferase reporter assays and a cell-based model of methamphetamine exposure also showed that miR-181a directly regulates expression of GRIA2. This study supports the evidence that miR-181a and the glutamate AMPA receptor gene GRIA2 play a critical role in methamphetamine-use disorder.

Methamphetamine-use disorder (MUD) is a major social and health concern. Almost 34 million people in the world had used methamphetamine (MA) at some time in their lives by the end of 2012^1^. It is estimated that approximately 57.1% of registered drug abusers in China suffer from MA abuse or dependence^2^. Because the understanding of the precise molecular mechanisms of MUD is limited, effective pharmaceutical therapies for MUD are still lacking. There is an urgent need to improve our knowledge of MUD and to develop novel therapeutic drugs for this complex and intractable disorder.

There have been attempts to elucidate the molecular pathways involved in MUD. Two important signal molecules related to drug-use disorders have been identified: glutamate ionotropic receptor AMPA type subunit 2 (GRIA2)^3^,^4^,^5^ and gamma-aminobutyric acid type-A receptor alpha1 subunit (GABRA1)^6^,^7^.

GRIA2 is a subtype of the glutamate AMPA receptor and is also the most abundant glutamate receptor in the central nervous system^8^. Moreover, compared to wild-type mice, GRIA2 knockout mice display impairment in learning stimulus-reward associations and deficits in conditioned place preference (CPP) to reward, anomalies known to contribute to aberrant addictive behaviours^9^. Blocking of GRIA2 via the selective glutamate AMPA receptor antagonist 6-cyano-7-nitroquinoxalone-2,3-dione (CNQX) can prevent the induction of an amphetamine-induced CPP^10^. However, there are no published clinical studies of the difference in the expression of GRAI2 between MUD patients and healthy controls.

GABRA1, a subunit of GABA type-A receptor, is believed to be a critical molecular switch in MUD. A preclinical study by our group revealed that the expression of GABRA1 in the dorsal striatum is significantly down-regulated in rats treated with MA^11^. Injection into the dorsal striatum of either the GABA type-A receptor agonist or the specific type-A receptor alpha1 subunit agonist significantly decreases MA CPP formation.

MicroRNAs (miRNAs) are endogenous small, noncoding RNAs that act as post-transcriptional regulators. Through base-pair interactions with the 3′-untranslated regions (3′-UTRs) of their target mRNA transcripts, miRNAs inhibit gene function by either repressing translation or inducing mRNA degradation^12^. Recent studies have shown that cocaine, nicotine and morphine exposure up- or down-regulate the expression of miRNAs^13^,^14^,^15^, and aberrant expression of miRNAs plays an important role in drug-use disorders. However, the roles of miRNAs in MUD are still unclear. To elucidate the roles of miRNAs in MUD and the mechanisms involved, we used miRNA microarray profiling and real-time qRT-PCR and found a significant difference in the miR-181a serum level between MUD patients and healthy controls. Additionally, chronic MA use inhibited the expression of miR-181a^16^. The 3′-UTRs of GRIA2 and GABRA1 mRNA are predicted (PicTar, TargetScan) to have miR-181a-binding sites.

In this study, we hypothesized that MA abuse alters the expression of miR-181a and that GRIA2 and/or GABRA1 expression may altered by aberrant expression of miR-181a via interactions at the 3′-UTR. These receptors may therefore jointly contribute to MUD. To test this hypothesis, first, we measured GRIA2 and GABRA1 expression in MUD patients. Second, we examined the effects of MA exposure on the expression of GRIA2 and GABRA1 *in vitro*. We further demonstrate that miR-181a plays an important role in MUD through the GRIA2 and/or GABRA1 pathway.

## Materials and Methods

### Participants

One hundred and twenty-four MUD patients were recruited from a compulsory rehabilitation centre in Shanghai. Eligible participants were required to meet the DSM-IV criteria for methamphetamine abuse or dependence; to be 18–65 years old; and to have no comorbid severe medical conditions, such as brain disease and cardiovascular disease. Patients who had used any substance other than nicotine or alcohol in a social setting were excluded. In this case-control study, subjects were diagnosed by two or more trained psychiatrists using the MINI, a simple, structured diagnostic interview designed to provide DSM-IV diagnoses of axis-I psychiatric disorders. The patients met the lifetime DSM-IV diagnostic criteria for methamphetamine abuse or dependence based on a structured interview.

Fifty-seven gender- and age-matched healthy controls were enrolled. The healthy controls had no self-reported family history of psychiatric conditions or history of drug use.

All participants were Han Chinese. This study was approved by the ethical committee of Shanghai Mental Health Center (approval number: 2011–28), and signed informed consent forms were obtained from all the subjects. The experiments were performed according to the regulations and guidelines established by this committee.

### Blood sample collection and ELISA measure

Blood samples were taken upon admission between 08:00 and 10:00 to minimize a possible circadian rhythm-variance bias. Ten millilitres of blood was collected, and the blood was immediately centrifuged at 3500 rpm for 10 min. The serum was stored at −80 °C until it was thawed for use in the assay. The serum levels of GRIA2 and GABRA1 were assessed using ELISA kits (catalogue numbers F00863 and F00938, Westang Bio-tech, Shanghai, China). All assays were performed according to the manufacturer’s directions and were performed in duplicate and expressed in pg/ml and ng/ml. The intra-assay and inter-assay coefficients of variation were 5.0 and 8.7%, respectively.

### SH-SY5Y cells culture and MA exposure

SH-SY5Y cells were obtained from the Committee on Type Culture Collection of Chinese Academy of Sciences (CTCC of CAS, Shanghai, China). SH-SY5Y cells were cultured in complete Dulbecco’s modified Eagle’s medium/Ham’s F12 (DMEM/F12 50:50 mix, Gibco, Carlsbad, California, USA) supplemented with 10% FBS and 2% penicillin-streptomycin. The cells were maintained at 37 °C in a 5% CO_2_ atmosphere and were seeded at 2 × 10^5^ cells per well in 96-multiwell plates and allowed to grow to 80% confluence.

Methamphetamine (MA) (purity > 99.1%) was obtained from the Shanghai Institute of Materia Medica, Chinese Academy of Sciences (Shanghai, China). MA treatments were performed as described by Chen *et al*.^17^. We divided the SH-SY5Y cells into a 2-mM MA group and blank group, which were exposed to 2 mM MA or no MA, respectively. After a 48 h MA treatment, total RNA or protein was isolated from the cells.

### RNA extraction and quantitative reverse-transcription PCR (qRT-PCR)

Total RNA was extracted from SH-SY5Y cells using the miRNeasy Mini Kit (Qiagen, Valencia, California, USA) and first-strand cDNA was synthesized using QuantiTect Reverse Transcription Kit (Qiagen, Valencia, California, USA). The qRT-PCR was performed on an ABI Prism 7900 Sequence Detection System (Applied Biosystems, Foster City, California, USA) based on the SYBR Green method, according to the instructions of the QuantiFast SYBR Green RT-PCR Kit (Qiagen, Valencia, California, USA). The levels of GRIA2 and GABRA1 RNA were calculated relative to GAPDH RNA (see Supplementary Material for more details).

### Western blot

Protein was extracted from SH-SY5Y cells using RIPA Lysis buffer (Life Technologies, Carlsbad, California, USA). Antibodies against GRIA2, GABRA1 and GAPDH were purchased from Abcam (Cambridge, Massachusetts, USA). Western blotting was performed according to the instructions of the Odyssey Western Blotting Kit (LI-COR Biosciences, Lincoln, Nebraska, USA) (see Supplementary Material for more details).

### Vector structure

The psiCHECK-2-GRIA2 and psiCHECK-2-GABRA1 vector, which expresses the full length cDNA for GRIA2 (Refseq NM_000826.3) and GABRA1 (Refseq NM_001127643), was purchased from Generay Biotech (Shanghai, China). The vector was used in dual luciferase reporter-gene assays in HEK 293T cells. The resultant constructs were sequenced to confirm their identity.

### HEK 293T and dual luciferase reporter gene assays

Human embryonic kidney 293T cells were obtained from the Committee on Type Culture Collection of Chinese Academy of Sciences (CTCC of CAS, Shanghai, China). 293T cells were used to test the dual luciferase reporter-gene assays. All media and supplements for cell culture were obtained from Life Technologies (Carlsbad, California, USA).

The 293T cells were maintained in Dulbecco’s Modified Eagle medium (DMEM) supplemented with 10% foetal bovine serum (FBS) and 2% penicillin-streptomycin. The cells were maintained at 37 °C in a 5% CO_2_ atmosphere and were seeded at 2 × 10^5^ cells per well in 96-multiwell plates and allowed to grow to 80% confluence.

After they reached approximately 80% confluence, cells were transfected with psiCHECK-2 vector (100 ng/well), together with miR-181a mimic (final concentration: 50 nmol/L), miR-181a inhibitors, or miRNA negative control (final concentration: 50 nmol/L) (Gene Pharma, Shanghai, China), using the Lipofectamine 3000 reagent (Life Technologies, Carlsbad, California, USA). Transfection efficiencies were normalized to the activity of Renilla luciferase expressed by co-transfection with 1 ng/well vector plasmid. The 293T cells were cultured 48 h after transfection. Firefly and Renilla luciferase activities were measured using the Dual Luciferase Reporter 1000 Assay System (Promega, Madison, USA). For luciferase assays, each assay was carried out in at least three independent experiments.

### Statistical analyses

For qRT-PCR analysis, SDS files were imported into Applied Biosystems RQ Manager Software, and automatic baseline and cycle threshold (CT) of 0.2 settings were used. The ΔΔCT method was used for GRIA2 and GABRA1 mRNA quantification. Data analysis and statistical analysis were performed using the −ΔΔCT, which is mathematically equivalent to the Log_2_RQ (relative quantification, also known as “fold-control”) value. Quantitative analyses for western blotting were carried out using the Odyssey CLx Infrared Imaging System.

Student’s *t*-tests were used to compare the differences in GRIA2 and GABRA1 expression between the two groups. All data were analysed in SPSS 16.0. Graphs were drawn using GraphPad Prism5. A two-tailed *p*-value of less than 0.05 was considered statistically significant.

## Results

### GRIA2 and GABRA1 expression of MUD patients

The demographic data of the MUD group and healthy controls are shown in Table 1. There were no significant differences in age, gender and years of education between the MUD patients and the healthy controls (*p* = 0.67, 0.55, 0.22).

The serum levels of GRIA2 and GABRA1 are shown in Table 1. The results indicated that there are differences in serum GRIA2 levels between the patient group and the healthy control group (*t* = 5.18, *p* < 0.01). The mean GRIA2 serum level in MUD patients was significantly higher than in the healthy controls (32.58 ± 10.46 pg/ml vs. 23.56 ± 9.68 pg/ml). Serum GABRA1 levels were significantly different between the MUD group and controls (1.09 ± 0.79 ng/ml vs. 1.57 ± 0.93 ng/ml, *p* < 0.05).

### MA exposure affects the expression of GRIA2 and GABRA1 *in vitro*

**Figure 1:**
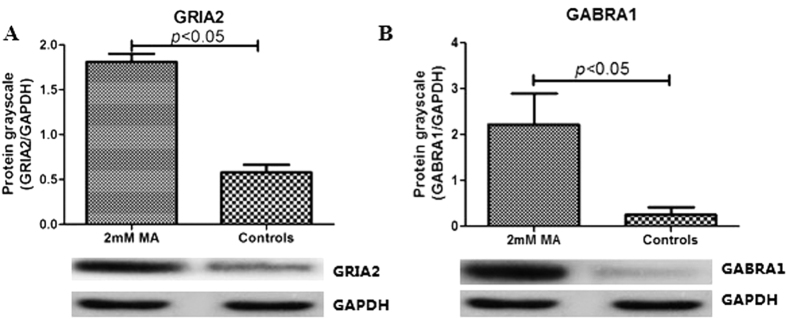
2 mM methamphetamine exposure up-regulate GRIA2 and GABRA1 protein expression in SH-SY5Y cells.

Reverse transcriptase PCR showed that exposure to 2 mM MA increased GRIA2 mRNA expression by 2.84 ± 1.57-fold compared to healthy controls (2.84 ± 1.57 vs. 1.01 ± 0.15 *p* < 0.01). Western-blot analysis was performed to assess whether the changes in mRNA expression were reflected in protein synthesis. GRIA2 protein also increased in SH-SY5Y cells following treatment with 2 mM MA (1.81 ± 0.09 vs. 0.16 ± 0.06, *p* < 0.05) (Fig. 1A), a finding consistent with the observed mRNA expression profile.

The RT-PCR assessment of mRNA also confirmed that relative expression of GABRA1 increased significantly after MA exposure (2.00 ± 0.98 vs. 1.08 ± 0.49, *p* < 0.05). Western blot analysis showed that the level pf GABRA1 protein was increased by treatment with 2 mM MA (2.23 ± 0.67 vs. 0.27 ± 0.15, *p* < 0.05) (Fig. 1B).

### miR-181a negatively regulates GRIA2 but not GABRA1

We used the dual luciferase reporter assay system (psiCHECK-2) to determine whether miR-181a interact with the 3′-UTR of GRIA2 and GABRA1 mRNA, thereby inhibited the reporter gene (luciferase) expression.

**Figure 2:**
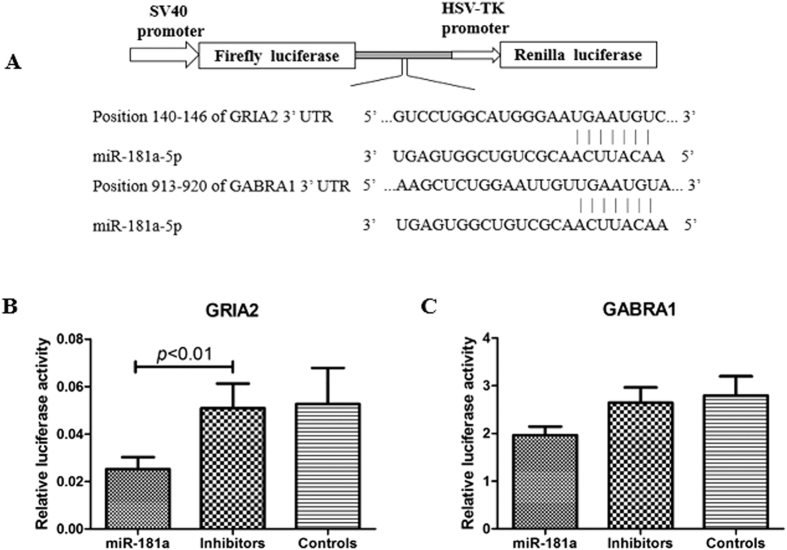
Luciferase reporter constructs and reporter gene assay. (**A**) the reporter gene vector (psiCHECK-2) containing the 3′-UTR of GRIA2 and GABRA1, showing one putative target site shared by miR-181a. (**B**) miR-181a inhibit GRIA2 expression. (**C**) miR-181a inhibit GABRA1 expression. Inhibitors, miR-181a inhibitors. Controls, miR-181a negative control.

In the recombinant reporter-gene construct, the GRIA2 and GABRA1 3′-UTR sequences spanning the predicted binding sites of miR-181a were positioned downstream of the firefly luciferase open reading frame in the psiCHECK-2 vector (Fig. 2A). Transfected miR-181 mimics negatively regulated reporter-gene expression in HEK 293T cell lines. miR-181a induced a reduction of more than 50% in luciferase activity at psiCHECK-2-GRIA2 vector and a reduction of more than 90% in luciferase activity at psiCHECK-2-GABRA1 vector (Fig. 2B,C).

## Discussion

In this study, we found that the GRIA2 levels of MUD patients were higher than those of healthy controls. More importantly, the results in MUD patients were consistent with those in the MA-exposure neural cell-based model. Glutamate receptors are divided into two groups according to their mechanism of action, as ionotropic and metabotropic glutamate receptors^18^. Ionotropic glutamate receptors mediated the vast majority of excitatory neurotransmission in the brain. The GRIA2 gene encodes glutamate AMPA ionotropic receptor 2, and previous findings have implicated GRIA2 in various drug use-disorders^19^,^20^ and drug-induced learning and memory impairment^21^. Mead and colleagues found that GRIA2 knockout mice display impairments in learning amphetamine-reward associations^21^. However, there is little research on how MA affects GRIA2 expression. To our knowledge, this is the first study to demonstrate these changes in the serum GRIA2 levels of MUD patients and in a MA-exposure neural cell-based model. The results revealed that GRIA2 should be further investigated as a possible target of treatment for MUD.

Moreover, we also found that miR-181a significantly down-regulated the expression of GRIA2 and inhibited the GRIA2 protein activity. Recent studies have demonstrated that the miRNA regulation of gene expression plays a crucial role in potentiating the functions of addictive drugs^22^,^23^,^24^,^25^. He and colleagues built a morphine-exposure SH-SY5Y cell-based model and identified the negative regulation of the μ opioid receptor (MOR) by let-7^26^. Their results suggest that let-7 plays an integral role in opioid tolerance. Another group found that cocaine injection results in an increase in miR-132 in the rat hippocampus and rapidly induces regulatory activity of CREB, which is an important signal in the cocaine-dependence pathway^27^. Researchers also assessed the expression profile of miR124a in the dorsal striatum of rats exposed to chronic alcohol intake and found that miR124a was down-regulated in that region^28^. They also found that brain-derived neurotrophic factor (BDNF) was up-regulated by miR-124a activity. Based on their study, we deduced that striatal miR124a and BDNF signalling have crucial roles in alcohol consumption and dependence. As mentioned above, most previous research has focused on morphine, cocaine and alcohol. Before our study, only one group had found that MA affected expression of miR-9 using frontal-cortex autopsy tissues from HIV-positive MA abusers^29^. In this study, we demonstrated that the regulation of GRIA2 expression by miR-181a plays a crucial role in MUD. Our data provide grounds for further research.

In addition, we found that MA use inhibited GABRA1 expression but miR-181a cannot directly negatively regulate the expression of GABR1. GABA receptors are the main inhibitory receptors in the central nervous system, and GABA-A receptor alpha1 subunit has the most important rapid central inhibitory effect^30^. Previous studies found that alcohol, a central inhibitor, increases the expression of GABRA1^31^,^32^,^33^. In our previous research, we found that the expression level of GABRA1 was significantly decreased in the dorsal striatum after conditioned methamphetamine pairing^11^. From our clinical study, we also found that the expression of GABRA1 is decreased among MUD patients. Unfortunately, these results are not consistent with the result obtained from the neural cell-based model of MA exposure. We could find nothing in the literature to help us to explain this finding. We should be cautious about drawing conclusions based on analyses of these data. More studies are needed to examine the association between GABRA1 and MUD.

The present study was limited in the following ways. First, we investigated the serum levels of GRIA2 and GABRA1 in only 124 MUD patients. Owing to the small sample size, the results must be considered preliminary. To verify the results presented here, larger samples will need to be recruited in a future study. Second, we measured the GRIA2 and GABRA1 levels in the serum rather than in the brain. However, previous studies have shown that serum levels reflect changes in brain-tissue neurotrophins^34^. Our study was a pilot study, we will verify the relationship between the serum and central nervous level of miR-181a and GRIA2 in our future study by PET and IPS cells.

In summary, our results show that the serum GRIA2 levels in MUD patients are higher than those in healthy controls, but GABRA1 expression decreased after MA use. In addition, we used dual luciferase reporter-gene assays and a cell-based model of MA exposure and found that miR-181a could negatively regulate the GRIA2 gene. MA abuse produces pathological changes to the brain that can endure even after long-term abstinence. In this context, our study provides a unique perspective shedding light on the role of miR-181a and post-transcriptional control GRIA2 on molecular adaptation to MA. The results of our study support the notion that miR-181a-GRIA2 pathway might play a critical role in MUD. Our results revealed that more research is needed to examine miR-181a and GRIA2 as potential targets of pharmaceutical therapy for MUD.

## Additional Information

**How to cite this article**: Zhang, K. *et al*. miR-181a is a negative regulator of GRIA2 in methamphetamine-use disorder. *Sci. Rep.* **6**, 35691; doi: 10.1038/srep35691 (2016).

## Supplementary Material

Supplementary Information

## Figures and Tables

**Table 1:** Demographic data and receptor gene expression of patients and healthy controls.

	Patients (n = 124)	Controls (n = 57)	*t/χ*^*2*^	*p*
Age (years)	36.65 ± 9.74	36.19 ± 10.60	−0.43	0.67
Male	84 (67.74%)	36 (63.16%)	0.37	0.55
Education (years)	9.74 ± 1.75	10.19 ± 1.96	−1.26	0.22
Age of initial use (years)	31.87 ± 10.77			
Duration of use (months)	22.21 ± 31.73			
Average dose one time (g)	0.44 ± 0.39			
GRIA2 (pg/ml)	32.58 ± 10.46	23.56 ± 9.68	5.18	0.00
GABRA1 (ng/ml)	1.09 ± 0.79	1.57 ± 0.93	−2.49	0.02

Patients: methamphetamine-use disorder patients; GRIA2: glutamate receptor, ionotropic, AMPA 2; GABRA1: gamma-aminobutyric acid type A receptor alpha1 subunit.
