# Supplementary material for: miR-181a is a negative regulator of GRIA2 in methamphetamine-use disorder
Source: Sci Rep. 2016 Oct 21;6:35691. doi: 10.1038/srep35691 (PMC5073328; doi:10.1038/srep35691)
Supplement: Supplementary Information [file srep35691-s1.docx]

**miR-181a is a negative regulator of GRIA2 in methamphetamine-use disorder**

Kai Zhang^a,b^, Qingzhong Wang^a^, Xuxiu Jing^a^, Yan Zhao^a^, Haifeng Jiang^a^, Jiang Du^a^, Shunying Yu^a*^, Min Zhao^a*^

a Collaborative Innovation Center for Brain Science, Shanghai Mental Health Center, Shanghai Jiao Tong University School of Medicine, 600 Wan Ping Nan Road, Shanghai 200030, China

b Wuxi Mental Health Center, Nanjing Medical University, 156 Qian Rong Road, Wuxi 214151, China

* Corresponding authors. Tel.: +86 21 34773299. E-mail addresses: yushuny@yahoo.com (S. Yu), drminzhao@gmail.com (M. Zhao).

1 Kai Zhang and Qingzhong Wang contributed equally to this work.

S1. The sequencing information of the 3'-UTR cloned vectors.

**GRIA2**

GGGATGACCTTGAATGATGCCATGAGGAACAAGGCAAGGCTGTCAATTACAGGAAGTACTGGAGAAAATGGACGTGTTATGACTCCAGAATTTCCCAAAGCAGTGCATGCTGTCCCTTACGTGAGTCCTGGCATGGGAATGAATGTCAGTGTGACTGATCTCTCGTGATTGATAAGAACCTTTTGAGTGCCTTACACAATGGTTTTCTTGTGTGTTTATTGTCAAAGTGGTGAGAGGCATCCAGTATCTTGAAGACTTTTCTTTCAGCCAAGAATTCTTAAATATGTGGAGTTCATCTTGAATTGTAAGGAATGATTAATTAAAACACAACATCTTTTTCTACTCGAGTTACAGACAAAGCGTGGTGGACATGCACAGCTAACATGGAAGTACTATAATTTACCTGAAGTCTTTGTACAGACAACAAACCTGTTTCTGCAGCCACTATTGTTAGTCTCTTGATTCATAATGACTTAAGCACACTTGACATCAACTGCATCAAGATGTGACATGTTTTATAAAAAAAGGAAAAAAAACATTTAAAACTAAAAAATATTTTTAGGTATTTTCACAAACAAACTGGCTTTTAAATAAATTTGCTTCCATATTGGTTGAATAAGACAAAAACAATTAAACTGAGTGGGAAGTGAATAAAAAAAGGCTTTAGGTATCGATTCCATATTTTTCAAAGCCAAATATGTAAATGCTAAGGAAAGTAAACAAAGAGGAGATTCCAATCTTGTAATTTAATATTGTTATTAAAACTTTAATGTATCCTATTCTTTAACATTTGGTGTTAATATAAAATTACTTGGCAATGCTTGACATTTGAAATAAACATTTTTCTATTGTTTTATTGCAAGTGGTCCAATTAATTTTGCTTAGCTACAGTTTGGTCATAAATCAAGTGAGTTTAAAGACACTACCAAGTTGTTAGGTGCCCAGAGAAAATTTCTCCCTTTTAAAAAGGCCAGGTGATTTTTCAAATGTAATCTTGCCCCCAAAGTAATATCTGAATATCTTTTTGACATGTCTAAATATATATATATATAAAGAAATATTTGTTAACACAAAAGCATTTGATCTATGTAGATAAATGCTAATAGATTTAAAAAGCTAATATTAACAAATACCAGAATACGTGAAGTTCCATTTTTAAAGTGTTTGAGCTTACAGAAGAGAAACATTCATTTTAAATGAAGTAAAAAATGCCTTGAAAGTAATTCTTTAGATAGTTGCCCATTGATTAAATTCCAAAAACTAAATATGTTTTTAGCTTTAAAATTATAAAAGCTGTCATAAACTTTATATATTATGAATTTTAAAATATGTTTGAGTCTCCTGCAATATAGTTTCATCCCATTGACATCAATTAAAAATAACCCTAATATATTATTTTTATATTTATTCCTCAGGTGGAATGGCTATTTTAATATGCCCAGTGTGGATAAAATGTCACATTTCTGTAACTTTTGACTAAAGAGCCTATATTTATCTAGTTAATGAATTTAAAGGATCTATCTTTCCCTTCATAAAATACCTCTTATTTCCATTAAAGCCCCCCAAGTTTAATTAATTTAGGATTTTGAATGATTATTGACATCCAATAGTTATTTTTAATATTTGTATTCTTGTTATTTCTGGAAGAAAGCCTTTGTGTAGCACTTGGTATTTTGCAAAGTGCTTTTAAAACATTCTTACTTACCGTATTTCATAGAAGGGAAGGAAAAATGTAAGGTTTAACAGTAAGCACTTGCATTGAACATGGAGGCATGTGGTATCATGATATTCTTCACTAAATTTAGCTGTCCCTAATCACAGATCCTAAGGTAATATAATATAATTTTAGTGCATTTCTCCTCATCAGGAATGCTGGAGGTGCATTTTAAGTTTTAATAATAAGTGCTAGAATGACCAAATTGCAGACTAATTGTTTCCATATTGTACTTAAAATGAGTTTTTAAAAGTGAAAAAGAAATGACTATATACAATCAATGCTATTTATTGTACCTCTGGGCCTACTCTTCTAAAAATTGTAGCTTATCGATTTTTCTCTGTCAAGCTTGAACTAATGTAAATAATTGAAATAATGTAAAGTTATATTTTCATGTTTTTATAGATACAACATGACAAGAATACATAATGTAAGAGTATTTCAACTATGGATAATGTTGATTGGATAATGCACATCTCAGTTACAAGCAGTACTCATAGTTTAATATCCATGTAACGGTGCATCAATATATTGCTATATAAATATGTCTGTGTGCATATAAGTGAAAAGTGGTCAAACAAGAGTGATGACAGCTGTCTAAAGGTTTTTTTATTCATTTTATATAAAAACTGTTATGGAAAGACCAAAATGTTTATGAACTATTCTTATGTAAATTTACAATTGTCCTTTACTGTACTTTTTTGTTTACAGTATAGTACCTTATTTTCTGCTGTGTTAAGTGGGTGTCAAACTCCAAGAAGACATACACTTTCTATAACTTCTATTGAAGATATTGGAATTTCCAATTTTTCATGTGTACTATGTCAGAAAATGCTTTCGATTTTATTTTTAAATCTAACATCGGATGGCTTTTCCGGAGTGTTGTAAAAACTTCAATCATACATAAAACATGTTCTTACAAAAGGCAAA

**GABRA1**

ATCTTTTACTCACATTCTGTTGTTCAGTCCTCTGCACTGGGAATTTATTTATGTTCTCAACGCAGTAATTCCCATCTGCTTTATTGCCTCTGTCTTAAAGAATTTGAAAGTTTCCTTATTTTCATAATTCATTTAAGAACAAGAGACCCCTGTCTGGCAGTCTGGAGCAAAGCAGACTATGCAGCTTGGAGACAGGATTCTGACAGAGCAAGCGAAAGAGCAAAGTCATGTCAGAAGGAGACAGAATGAGAGAGAAAAGAGGGGGAAGATGGTTCAAAGATACAAGAAAAAGTAGAAAAAAAAATAACACTTAACTAAAACCCCTAGGTCATTTGTAGATATATATTTCCAAATATTCTAAAAAAGATACTGTATATGTCAAAAATATTTTTATGTGAAGGTGTTTCAAAGGGTAAATTATAAATGTTTCATGAAGAAAAAATTTTAAAAATCTACGTCTTTATTACACAAACTATGGTGTGCTTATGTTTTTGTTTTGCTTTTTAAACTGATGTATAGCTTTAACATTTTGTTTCCAAAGCTGAAGATCCCCATTCTTTCTCTTTGAAAAAAAAAAAGGCCTAATGCATTATTTTGTCATAAAATGCTATTTTAAAATTCATGGAACTTTCATACGTAAAGGTGCAGTTGCTCATTGTAGAGCACATTTAGTCCAATGAAGATAAATGCTTTAAATAGTTTACTTCACTTTCATCTGAGCTTTTACCACTAGACTCAAGGAAGAATAATTTTAACAGACATGTATACTCCATAGAAACTAAACTAAAATAGTTTAAAAATATTCCCTTTTTCACCCTATTTTCAGATAGCACATGAGCCCAACACTCACTTAATTCTCATTATGAAGATGTTTTTAGAGGGGCAAAAATATTTTGCAAGCTCTGGAATTGTTGAATGTATTCTTTTATATAACTACATTAAAAGCTTTAGATTGAAATTTATGACTAGCAAACAAAAATAGAATATATAAACGATATATGTAAATATACAGCATGAGATTGTACATTTTTTACTTTTTTAAAATTGTGTTCTTAAAATATTGTGTAAGAATCACTGCACTTAGCTGTTGGAATGTTGTTAAATGCTATGGAAATACATTTAGAACCTGCATTTAAGAACAGAACAGCAAGTATGAACCACATGGAACTTAAAACATATGGGTGTGAAGTCCACTTATGTAGACAAAACTTATAATTTCCAAACTGTTGTCTAGTATACAGTGATCAGTTGCTCTCTGTTCAAGTCATTCCACACATTTCCCTATTTTAGGCTATTATAATATAGAAAGAAAATGGGAAGCATTAGTTGGAGCTAGAAAATGAACTGTATATTATTGCTATATTTGCTAATACCAACTATTTCAATAAGTGTTGTACCATATGTAGCATTAAATATAAAATACATAAAAGAATGTACAGAAAATAGCTTTTATTGAGTAATATTACATTTCATTTATACTGTAGCAATATATTTGTAGGTATACTATGTAAGGGCTTTAAATAAAAGAGGTCCATTAATACTTCCTTATAAAAATTCTAGTCTGTTTCATTACTGCCCAGATGTTTTAGAGATAAATATTTATGCAGAAGGTATTTTTGAAGTCTCCTTTTGTCTGATAGAGTTTAACAGATATTTAAATTTAGTGCTCAGAATCCACAAGTCACGGTCTAAACACACTTAGAATACTACAGCATAAATCTGTTAGCATTATTGCCAAATAAGACAGTTGGGATCCAAACCCAAGTCTTGAGCAATGTTTTTCTCAAAAAGCTGCTATCCAATGATATAGGAAAATACATTGTGTTTTCCTAAACACACTTTTCTTTTTAAATGTGCTTCATTGTTTGATTTGGTCCTGCCTAAATTTCACAAGCTAGGCCAATGAAGGCTGAATCAAAGACATTTCATCCACCAATATCATGTGTAGATATTATGTATAGAAAATAAAATAAATTATGGCTCTAACTTCTGTGTTGCTGTTTATCTTGTTATTTTTCGGCGTTATACTAATGTGTTTATTGAGAGCATTTTACCTTCCAGACTTCTCATGGCTAACTTTTGGTCTGTATTTTGCTCCTTAGATGTGAATATTTCTTATTAGTCTGCTTCCTGCTACGCAATGACTGCATTTCTATCATTTCTCAGTTTGTTAGTATATGTGGATAGTATTCTACTGTATAAATGATTGCAAAGTTTATCAAAAACAAATTATTATATGTAGCTTTTCTACAGTGCTTTGCTAAACCATGTAGTACTAGTTAAGTCTTCCTTGAAAATAAAGATACACTCTTATAGGGGACAGTTCCTGTTCACTCCCAGGAAACTTTTTTAAAAGATGACACTGAATGTTTATTGCACTTTAGTGCAGTGAAGTGGCAATAAAACCTAACATGAATCAAGGTTGTTTATGGCAGATGCATGTGTTGCTTTACAGAGTTTAGCAAAAGCTCTTAATTTTATGTCATACTGTATTCTACTGAATAATAAAGCTAACATTATTCAATAATAAAATGGAATACTTGACTCTCTTTTCATGTT

S2. Western Blotting

Cells were trypsinized and washed twice with PBS, pelleted then re-suspended in 100 µl of RIPA Lysis buffer (Life Technologies, Carlsbad, California, USA) (0.5% Nonidet P-40, 50 mM Tris, pH 7.8, 150mM NaCl with a protease inhibitor mixture dissolved in the lysis buffer). The cell and lysis buffer mixture was incubated on ice for 30 min, and then centrifuged in a refrigerated microfuge for 10 min at 20,800 rcf at 4°C. Protein levels were determined using a BCA assay (Thermo Fisher Scientific, Houston, Texas, USA). Equal amounts of protein were boiled in 2 µl of 10× Sample Reducing Agent (Invitrogen, Grand Island, New York, USA) and 5 µl 4× LDS Buffer (Invitrogen, Grand Island, New York, USA). Samples were then loaded onto a 4-12% gradient gel (Invitrogen, Grand Island, New York, USA), ran at 200V for 1 h and transferred onto nitrocellulose membranes using the wet blot method. The membrane was then blocked in Odyssey blocking buffer (diluted 1:1 with PBS), for one hour at room temperature. After blocking the membrane was probed with Anti-Ionotropic Glutamate receptor 2 antibody (ab52180) (Abcam Incorporated, Cambridge, Massachusetts, USA) or Anti-GABA A Receptor alpha 1 antibody (ab94585) diluted 1:500 in blocking buffer, and Anti-GAPDH antibody (ab8245) diluted 1:5000 in blocking buffer, and incubated O/N at 4 °C. Following incubation with primary antibody, the membrane was washed with 0.1% PBS-Tween wash buffer before probing with Odyssey secondary antibody diluted 1:20,000 for one hour at room temperature. The membrane was then washed in 0.01% PBS-tween before infrared scanning using the Odyssey LI-COR imaging system.

S3. RNA extraction and quantitative reverse-transcription PCR (qRT-PCR)

Cells were washed twice with PBS before adding 600 µl of buffer RLT from the Qiagen RNeasy kit (Qiagen, Valencia, California, USA) directly onto the monolayer of cells, and left to incubate for 5 min at room temperature. Post-incubation with buffer RLT, cells were scraped using a scraper and the cell/buffer RLT mix was then added to a QIAshredder column (Qiagen, Valencia, California, USA) and centrifuged following the manufacturer’s instructions to homogonize the sample. The Qiagen RNeasy protocol was then followed to extract RNA from the SH-SY5Y cells stably expressing GRIA2 and GABRA1. The DNA was removed using DNase treatment (Qiagen, Valencia, California, USA) on column. The cDNA was made using the QuantiTect Reverse Transcription Kit (Qiagen, Valencia, California, USA), following the protocol provided. To validate the GRIA2 and GABRA1 expressing, a SYBR Green protocol was followed QuantiFast SYBR Green RT-PCR Kit (Qiagen, Valencia, California, USA), using primers designed by Generay Biotech (Shanghai, China). Results were all normalized to a GAPDH endogenous control.
